# Case report: Tenosynovial giant cell tumor

**DOI:** 10.3389/fonc.2024.1445427

**Published:** 2024-09-26

**Authors:** Anke Fähnrich, Zhala Gasimova, Yamil Maluje, Fabian Ott, Helen Sievert, Stephanie Fliedner, Niklas Reimer, Axel Künstner, Niklas Gebauer, Maxim Kebenko, Nikolas von Bubnoff, Jutta Kirfel, Verena-Wilbeth Sailer, Christoph Röcken, Bjoern Konukiewitz, Wolfram Klapper, Alex Frydrychowicz, Sam Mogadas, Gerdt Huebner, Hauke Busch, Cyrus Khandanpour

**Affiliations:** ^1^ Medical Systems Biology Group, Lübeck Institute of Experimental Dermatology, University of Lübeck, Lübeck, Germany; ^2^ University Cancer Center Schleswig-Holstein, University Hospital of Schleswig- Holstein, Lübeck, Germany; ^3^ Department of Hematology and Oncology, University Hospital of Schleswig-Holstein, Lübeck, Germany; ^4^ Institut for Pathology, University Hospital of Schleswig-Holstein, Lübeck, Germany; ^5^ Department of Pathology, Christian-Albrechts-University, Kiel, Germany; ^6^ Institut for Haematopathology, Christian-Albrechts-University, Kiel, Germany; ^7^ Department of Radiology, University Hospital of Schleswig-Holstein, Lübeck, Germany; ^8^ Department of Internal Medicine III, Ameos Krankenhausgesellschaft Ostholstein, Eutin, Germany

**Keywords:** single cell sequencing (scRNA-seq), RNA sequencing (RNA-seq), CSF1 fusion transcript, tenosynovial giant cell tumor (TGCT), molecular tumor board (MTB)

## Abstract

Tenosynovial giant cell tumor (TGCT) is a rare type of tumor that originates from the synovium of joints and tendon sheaths. It is characterized by recurring genetic abnormalities, often involving the CSF1 gene. Common symptoms include pain and swelling, which are not specific to TGCT, so MRI and a pathological biopsy are needed for an accurate diagnosis. We report the case of a 45-year-old man who experienced painful swelling in his right hip for six months. Initially, this was diagnosed as Erdheim-Chester disease. However, whole exome sequencing (WES) and RNA-Sequencing revealed a CSF1::GAPDHP64 fusion, leading to a revised diagnosis of TGCT. The patient was treated with pegylated interferon and imatinib, which resulted in stable disease after three months. Single-cell transcriptome analysis identified seven distinct cell clusters, revealing that neoplastic cells expressing CSF1 attract macrophages. Analysis of ligand-receptor interactions showed significant communication between neoplastic cells and macrophages mediated by CSF1 and CSF1R. Our findings emphasize the importance of comprehensive molecular analysis in diagnosing and treating rare malignancies like TGCT.

## Introduction

Tenosynovial giant cell tumor (TGCT), previously called pigmented villonodular tenosynovitis (PVNS) or giant cell tumor of the tendon sheath, is a rare mesenchymal neoplasm arising from the synovium of joints and tendon sheaths. It is molecularly characterized by recurrent genomic aberrations often involving the colony-stimulating factor 1 gene (CSF1) (1, 2). According to the 2020 WHO Classification of Soft Tissue and Bone Tumors, TGCT is a type of neoplasm that is locally aggressive (3). The symptoms of TGCT, including pain, tenderness, swelling, or limitation of motion, are not specific. Therefore, it is necessary to perform magnetic resonance imaging (MRI) and pathological biopsy to make an accurate diagnosis (4). Localized TGCT is a focal mass that typically surrounds or abuts the tendon, while the diffuse type often causes joint effusion or synovial fluid (5, 6). Sometimes, TGCT can resemble other soft tissue tumors on MRI, making diagnosis challenging (7).

## Materials and methods

A bone marrow biopsy was obtained and immediately stored in a preservation buffer, Singleron, Germany. The cell suspension was then shipped overnight at 4°C to Singleron Labs in Cologne, Germany. Cell viability was assessed at Singleron Labs, and the sample with viability exceeding 80% were subsequently processed into single-cell suspensions using the sCelLiVE Tissue Dissociation Solution (#1190062, Singleron). Library preparation was carried out using the GEXSCOPE Single Cell RNA Library Kit on a microwell chip (SCOPE-chip) with barcoded beads. The finished library was paired-end sequenced on an Illumina NovaSeq.

### Single-cell RNA-seq analysis pipeline

Raw gene expression matrices were generated by a custom pipeline combining *‘kallisto’* (v.0.46.1) and *‘bustools’* (v.0.46.1) using GRCh38 as human reference The output-filtered gene expression matrices were analyzed in ‘R’ (v.4.2.1), where empty droplets and doublets were removed for each sample using the packages ‘DropletUtils’ (v.1.8.0) and ‘doubletFinder’, and further analysis was performed using the ‘Seurat’ (v.4.3 (8)) package. Low-quality cells were excluded; cells with fewer than 200 features or less than 10% mitochondrial RNA were removed. Cell clusters were manually annotated using established marker genes from cited literature, complemented by automated approaches, including scType (9), based on the Human Cell Atlas. To identify and visualize the cell cross-talk among cells or between clusters, the R package ‘CellChat’ (v.1) was used according to the developer’s vignette [ https://github.com/sqjin/CellChat]. To identify molecular pathways that are differentially regulated between moderate and severe PCS patients, the PROGENy database was used (10). The PROGENy database, incorporating empirical activity weights from perturbation experiments for perturbation response genes, was used to estimate the activity of 14 signaling pathways. The pathway activity is statistically tested between moderate and severe PCS groups by incorporating a linear model (10).

### Bulk RNA-sequencing

For RNA-sequencing, STAR ALIGNER (version 2.7.2b) were used. Additionally, fusion genes from RNA-seq data were identified by the application of STAR-FUSION (version 1.9.0) running in *de novo* reconstruction mode. The hg19 genome served as the reference genome. The validation of resulting fusion genes was performed by applying FUSIONCATCHER and FUSIONINSPECTOR.

## Case presentation

**Figure 1:**
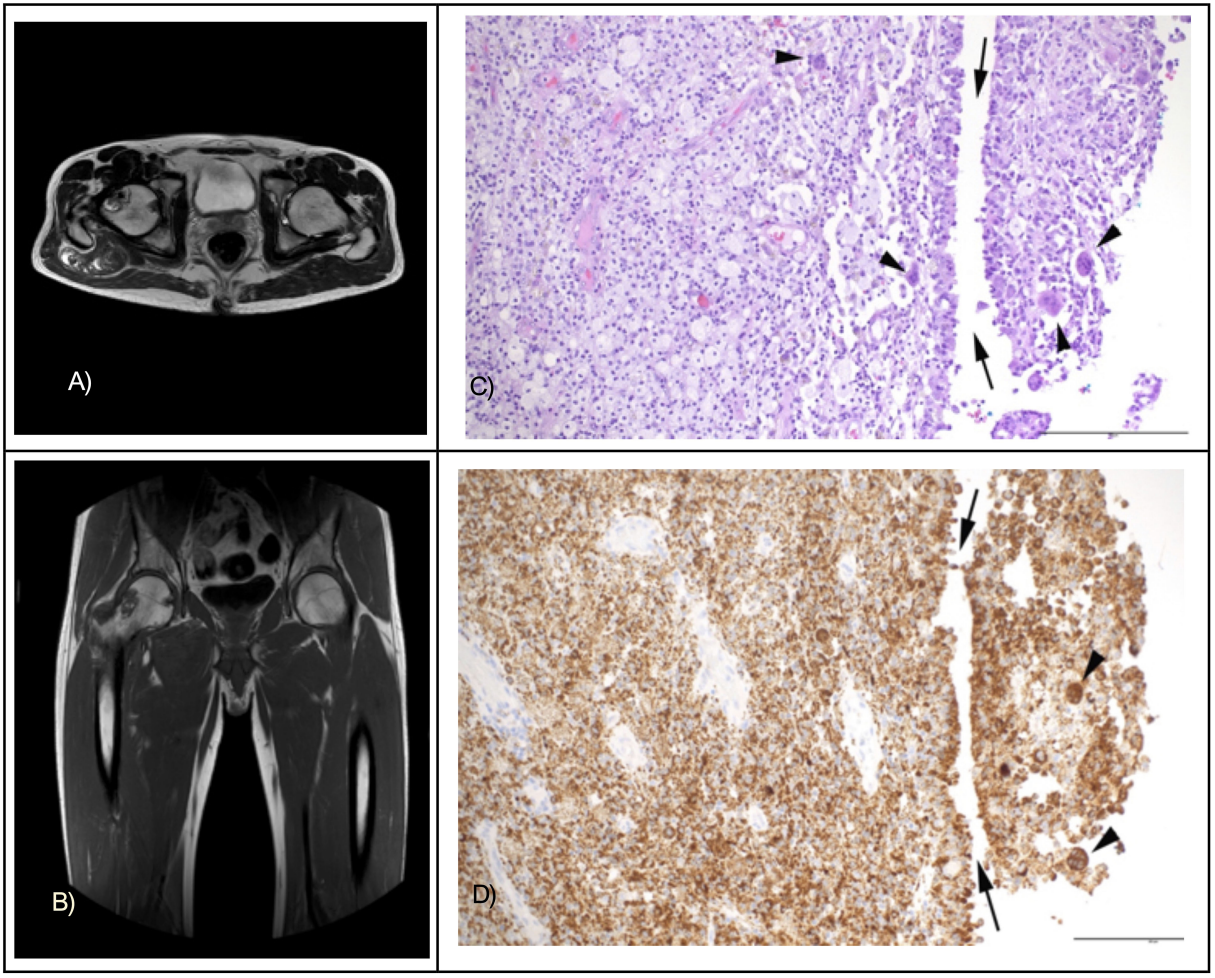
MRT images of the affected femoral head in the transverse plane **(A)** and frontal plane **(B)**. Tumor tissue with a predominance of infiltrating foamy macrophages, cleft-like spaces (arrows), and multinucleated giant cells (arrowheads) in hematoxylin and eosin stainings **(C)** and immunostaining for CD68 **(D)**. Original magnifications 200x, bar indicates 200 µm.

A 45-year-old male patient presented with a six-month history of progressively painful swelling in his right hip joint. Histological evaluation of a biopsy prompted a preliminary diagnosis of Erdheim-Chester disease. However, the clinical and radiological appearance were atypical, see **Figure 1**. The femoral head MRI scans (**Figures 1A, B**) reveal bone and soft tissue infiltration by the tumor from various perspectives. Additionally, **Figures 1C, D** present the tumor tissue, characterized by an abundance of foamy macrophages and multinucleated giant cells, as seen from the hematoxylin and eosin stainings (C) and immunostaining for CD 68 (D).

Due to the unclear diagnosis, the patient underwent whole exome sequencing (WES) of the primary tumor at the University-Cancer-Center-Schleswig-Holstein (UCCSH), using blood as a germline control. The results were presented at the local molecular tumor board (MTB). Variant calling excluded driver mutations in *KRAS*, *NRAS*, or *BRAF*, which would have been characteristic of Erdheim-Chester disease (11). Instead, WES identified 13 somatic mutations, two of which are known to be associated with cancer: a stopgain mutation (of unclear significance) in MAP2K2 p.Q218X and a recurrent nonsynonymous variant of uncertain significance in v p.C404Y (12). Further analysis of the mutational landscape found a low tumor mutational burden (0.85 mutations/MB) and microsatellite stability, with a notable deficiency in homologous recombination, scoring 66 (cut-off ≥ 42). Additionally, we assessed the BRCAness score based on the SBS6 tumor signature to predict responsiveness to PARP inhibitors but obtained a BRCAness score of 0.

Bulk RNA sequencing (RNA-seq) of the tumor tissue identified a CSF1::GAPDHP64 (1:109925202:: 1:116720422) fusion, a genomic aberration frequently found in TGCT. Based on the above evidence, the patient was re-diagnosed with TGCT driven by a CSFR1 fusion protein with characteristics of a histiocytic disorder. A surgical removal was not possible without large-scale mutilation. As diagnostic outcome from WES and RNA-seq analysis the following treatment options were proposed in decreasing preference: (i) Initiating interferon therapy (ii) administering pexidartinib, a tyrosine kinase inhibitor targeting the CSF1R pathway (iii) implementing therapy targeting MEK2 inhibition (iv) utilizing a tyrosine kinase inhibitor like imatinib (v) considering PARP inhibition supported by the pathological HRD score.

Based on the MTB recommendations, the patient was first treated with pegylated interferon. Six months into the interferon therapy, a restaging revealed a minimal reduction of the gluteal tumor mass and an increase in cystic tumor components. The next therapeutic option under consideration was the utilization of pexidartinib for the CSFR1 pathway. However, pexidartinib is not yet approved and therefore unavailable in the EU. Instead, therapy proceeded using imatinib, resulting in stable disease after 3 months.

**Figure 2:**
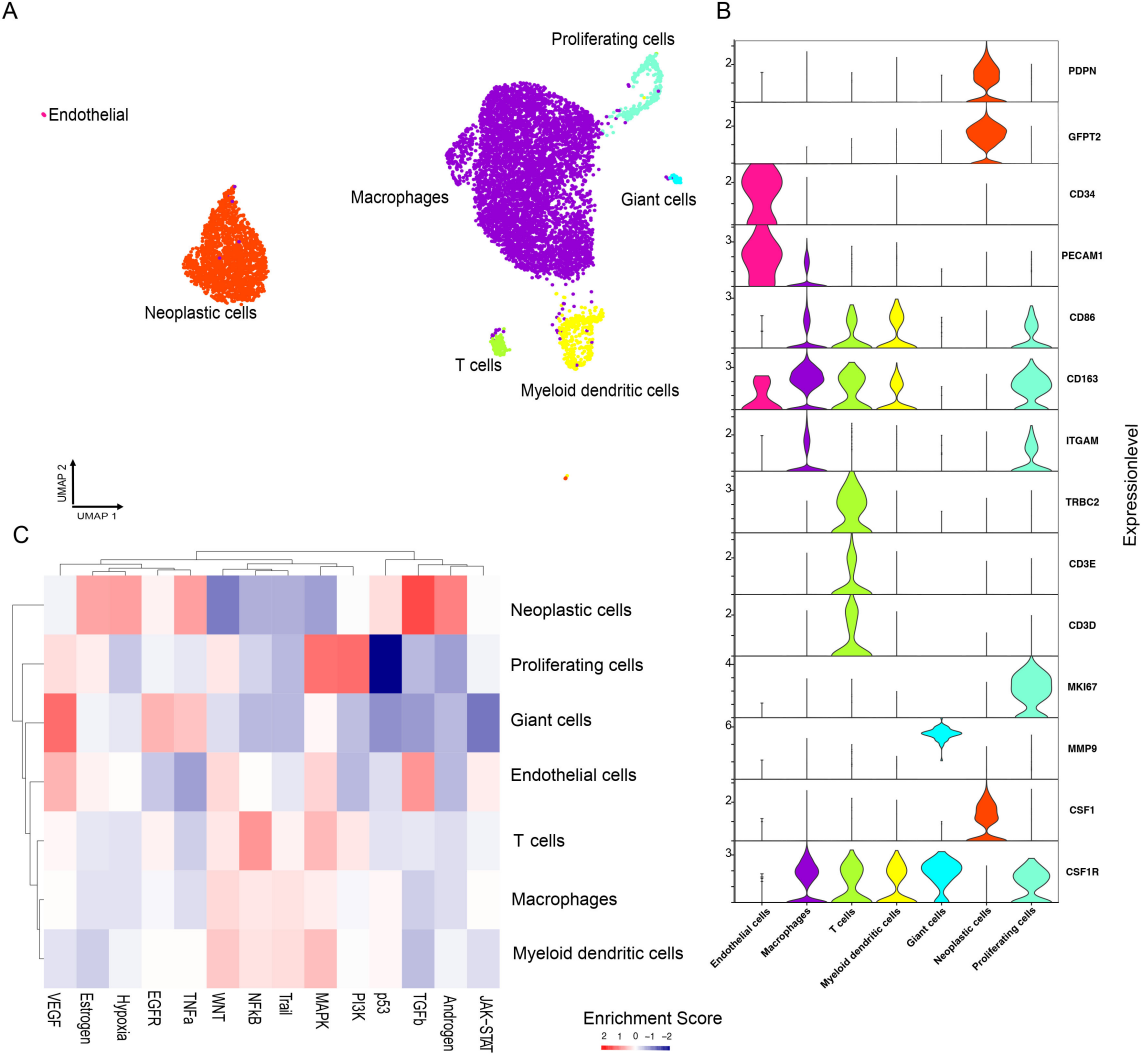
scRNA-seq analysis of cellular composition of bone marrow from Tenosynovial Giant Cell Tumor patient. Single-cell suspension from tenosynovial giant cell tumor patient analyzed by scRNA-seq. **(A)** UMAP plot of seven distinct cell clusters. **(B)** Violin plots of marker gene expression for the seven distinct clusters. **(C)** Relative pathway enrichment. Red indicates positive, whilst blue indicates negative enrichment.

Given the low incidence of TGCT, an understanding of tumor heterogeneity and its interaction with the microenvironment remains elusive. We, therefore, performed a single-cell transcriptome analysis of a tumor biopsy from the bone marrow. A total of 8,100 cells were obtained from one patient diagnosed with TGCT. Dimension reduction by Uniform Manifold Approximation And Projection (UMAP) of the gene expression matrix identified seven unique cell clusters (**Figure 2A**), which were identified as macrophages, T cells, proliferating cells, endothelial cells, myeloid dendritic cells, neoplastic cells, and giant cells. The marker genes of each cluster were: *CD86*, *CD163* for macrophages; *GFPT2*, *PDPN* for neoplastic cells; *CD3E*, *CD3D*, *TRBC2* for T cells; *PECAM1*, *CD34* for endothelial cells, *MMP-9* for giant cells (**Figure 2B**). The expression patterns of all seven clusters were analyzed to elucidate the location and expression of *CSF1* and *CSF1R*. **Figure 2B** shows that *CSF1* is exclusively expressed in the neoplastic cell type; and *CSF1R* is mainly found in macrophages, dendritic cells, T cells, and giant cells. These findings imply potential communication pathways linking neoplastic cell, dendritic cells, and T cells with macrophages. Previously, van IJzendoorn et al., 2022, identified a subset of tumor cells bearing a CSF1 translocation, exhibiting heightened CSF1 levels (13). High expression of *CSF1* in this patient is restricted to neoplastic cells. Neoplastic cells highly expressing *CSF1* attract and induce the proliferation of monocytes that express the receptor for CSF1 (CSF1R) on their surface (14). In our study *CSF1R* is highly expressed in the giant cells, and macrophages, but is absent from the neoplastic cells and other cell populations. Our study also observed that a major component of cells in TGCT consists of bystander macrophages responding to CSF1, **Figure 2B**.

The neoplastic cells have a significant impact on their surrounding environment by attracting a high number of macrophages. This is referred to as the “landscaping effect”. It is worth noting that CSF1R, which plays a crucial role in the growth of cells, is predominantly present in macrophages, dendritic cells, and T cells. Ligand-receptor interactions were investigated within neoplastic cells and other cell types using CellPhoneDB (15), revealing notably robust interactions between neoplastic cells and macrophages, while also revealing significant, albeit less pronounced interactions with giant cells, T cells, and myeloid dendritic cells mediated by CSF1 and CSF1R (**Supplementary Figure S1**). Our findings align with prior research, indicating that there are no direct CSF1 interactions with neoplastic cells, implying the lack of stimulation of neoplastic cells in TGCT through secreted CSF1 via an autocrine loop.

The putative upstream signaling pathways per cell type were assessed using PROGENy, which correlates known gene response patterns from upstream pathway activities with the observed gene expression. We observed a positive enrichment of pathways mainly in proliferating and platelet cells, comprising inflammatory signaling pathways (TGF-β), PI3K pathway, and MAP kinase signaling. Conversely, cell proliferation, linked to p53 signaling, exhibited negative enrichment, **Figure 2C**.

In summary, we report on a patient initially diagnosed with Erdheim-Chester disease. RNA-seq identified a CSF1 fusion, characteristic of TGCTs, leading to the correct diagnosis of the patient’s tumor. The TGCT literature predominantly consists of genetic studies, clinical case reports, and treatment strategies, yet precise analysis of its cellular heterogeneity remains challenging. To address this, our study conducted a comprehensive single-cell transcriptome analysis on a TGCT patient. The scRNA-seq analysis unveiled the complex cellular landscape and intratumoral diversity. Examination of all seven clusters indicated a predominant expression of CSF1 in neoplastic cells. Previously, Van IJzendoorn et al. (2022) identified tumor cells with heightened CSF1 levels due to a CSF1 translocation, attracting and stimulating monocytes expressing CSF1R (13). In our study, CSF1R was highly expressed in giant cells and macrophages, but absent in neoplastic cells. Bystander macrophages responding to CSF1 constitute a significant portion of cells in TGCTs, termed the “landscaping effect”. Ligand-receptor interactions investigated via CellPhoneDB showed robust interactions between neoplastic cells and macrophages, implying no direct CSF1 stimulation of neoplastic cells which is in line with previous findings. However, in atypical TGCTs, fusions were identified that are not associated with CSF1, indicating the presence of alternative pathogenic mechanisms that do not involve CSF1 (16).

In conclusion, extensive analysis by the molecular tumor board enabled here the final diagnosis and discovers new potential targets for this patient and MTB could be an avenue to diagnose and guide therapy for rare malignant diseases.

## Data Availability

The original contributions presented in the study are included in the article/**Supplementary Material**. Further inquiries can be directed to the corresponding author.

## Supplementary material

Supplementary Figure 1 Ligand–receptor interactions for the neoplastic cells in TGCT. CellphoneDB identified interactions involving CSF1, between the neoplastic cell clusters and other cell clusters.
