# Supplementary figures and images for: Case report: Tenosynovial giant cell tumor
Source: Front Oncol. 2024 Sep 26;14:1445427. doi: 10.3389/fonc.2024.1445427 (PMC11464255; doi:10.3389/fonc.2024.1445427)

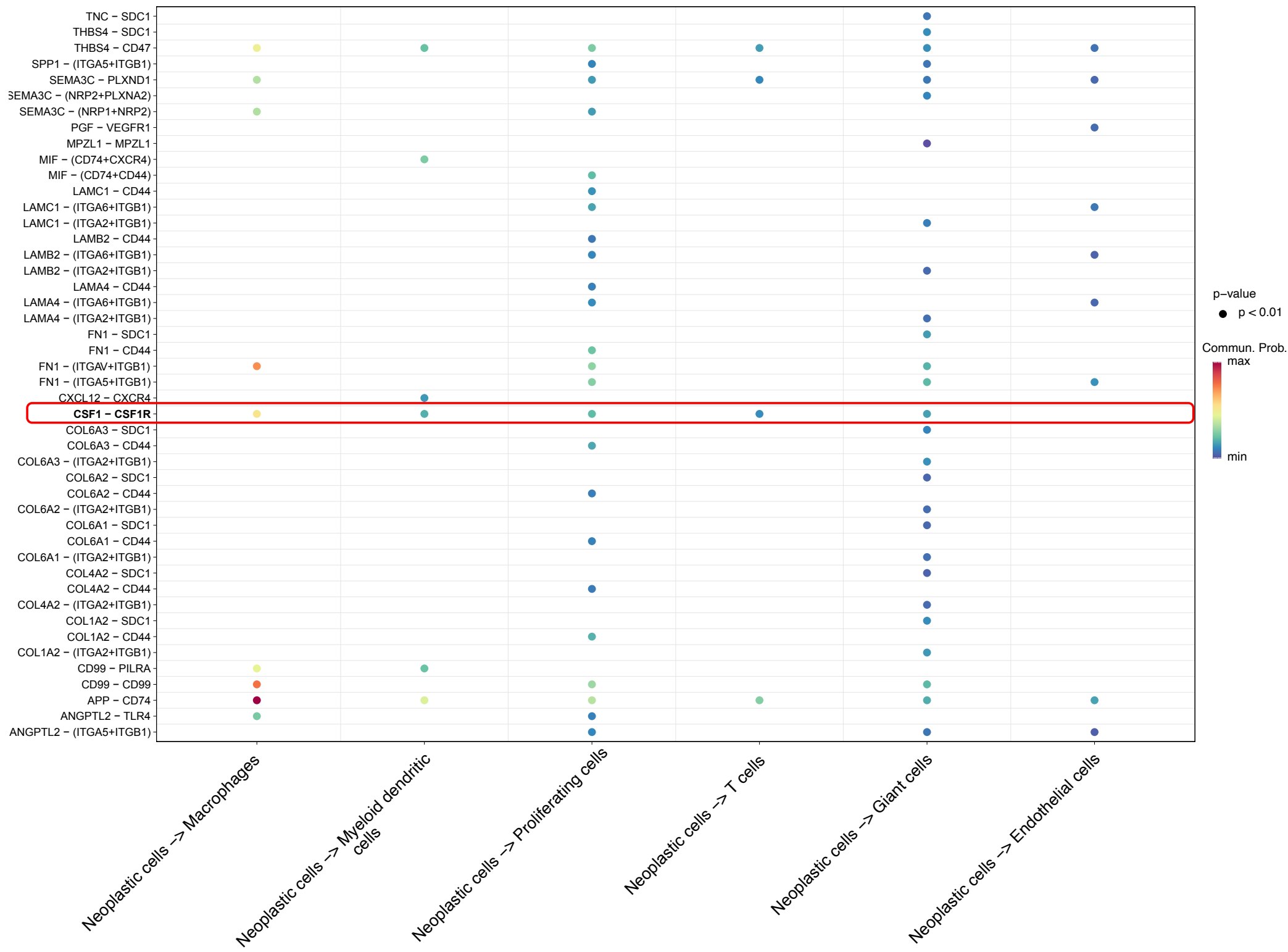

Supplement: Supplementary Figure 1 — Ligand–receptor interactions for the neoplastic cells in TGCT. CellphoneDB identified interactions involving CSF1, between the neoplastic cell clusters and other cell clusters. [file DataSheet1.pdf]
